# Efficacy of *Evolvulus alsinoides* (L.) L. on insulin and antioxidants activity in pancreas of streptozotocin induced diabetic rats

**DOI:** 10.1186/2251-6581-12-39

**Published:** 2013-07-08

**Authors:** Duraisamy Gomathi, Ganesan Ravikumar, Manokaran Kalaiselvi, Kanakasabapathi Devaki, Chandrasekar Uma

**Affiliations:** 1 Department of Biochemistry, Karpagam University, Coimbatore, 641 021 India; 2 Hawasaa University, Hawasaa, Ethiopia

**Keywords:** Streptozotocin, *Evolvulus alsinoides*, Pancreas, Antioxidant activity, Histopathological analysis

## Abstract

**Aim:**

Diabetes mellitus (DM), a leading non communicable disease with multiple etiologies is considered as third greatest cause of death in all over the world. During DM, persistent hyperglycemia causes an increased production of free radicals via auto oxidation of glucose and non-enzymatic protein glycation which may lead to disruption of cellular functions and oxidative damage to membranes. The present study was designed to investigate the therapeutic effect of *Evolvulus alsinoides* on antioxidant activity in pancreas of experimental diabetes.

**Methods:**

The antioxidant activities were done by using standard protocols. For histopathological analysis, the pancreatic tissues of all experimental groups were fixed with 10% formalin for 24 hrs then the samples were stained with haemotoxylin-eosin for the microscopic observation.

**Results:**

Oral administration of plant extract for 45 days resulted in significant antioxidant activity, increases the insulin level and also inhibits lipid peroxidation in pancreas of streptozotocin induced diabetic rats. The histopathological studies showed the normal histology of pancreas after treatment with plant extract and glibenclamide. This study showed that the administration of *Evolvulus alsinoides* to streptozotocin induced diabetic rats improves the antioxidant activity and remodel the structure of pancreas due to the presence of secondary metabolites like phenols, flavonoids, alkaloids, steroids, terpenoids and glycosides in the ethanolic extract of plant material.

**Conclusion:**

From the present study, it can be concluded that the plant extract effectively reduced the oxidative stress induced by streptozotocin and potentially increased the insulin level. Hence, it can be used in the management of diabetes mellitus.

**Electronic supplementary material:**

The online version of this article (doi:10.1186/2251-6581-12-39) contains supplementary material, which is available to authorized users.

## Introduction

In diabetes mellitus, chronic hyperglycaemia produces multiple biochemical sequelae and diabetes-induced oxidative stress that play an important role in the symptoms and progression of the disease [1]. Free radicals have been concerned in the causation of several disorders which includes diabetes and several other disorders [2, 3]. Increased oxidative stress has been postulated in the diabetic state which coexists with a reduction in the antioxidant status [4]. Tissue antioxidant status has altered in diabetes resulting in increased oxidative damage of membranes and tissue injury [5–7].

Antioxidants may have great potential in ameliorating these disease processes and play an important role in protecting the human body against oxidative damage by free radicals [8]. Supplementation of therapeutics with antioxidants may have a chemo protective role in the diabetes [9]. Present therapeutic strategies mostly try to relieve the clinical manifestation of diabetes and complications. Since diabetes seems to be a stress - related disorder and the antioxidants may be useful in preventing the disorder. Many plant extracts and its products have been shown to have significant antioxidant effect in treating these kinds of diseases [10–13]. Activity of important antioxidant enzymes like glutathione peroxidase and glutathione reductase, that directly scavenge free radicals or prevent their conversion to toxic products [14], is also altered in diabetic condition [5]. The control of different metabolic derangements is needed to prevent the development of diabetes and its complications [15, 16].

Report of ethanobotany suggested that about 800 medicinal plants possess antidiabetic potential and the bioactive compounds such as glycosides, alkaloids, terpenoids and flavonoids (phenols) are effective drugs both in preclinical and clinical studies [17, 18]. *Evolvulus alsinoides* is a perennial herb belonging to the family of Convolvulaceae with a small woody and branched root stock. The plant is widely distributed in tropical and subtropical regions throughout the world. It grows commonly as a weed in open and grassy places throughout India, ascending to 6,000 fit. The herb was used to treat dysentery and to treat fever. Mohammedan physicians used the plant as a general tonic to strengthen the brain and memory. It was used to treat bowel problems and to promote conception. The entire plant was considered astringent and useful for treating hemorrhages and there are a variety of other medical applications, including as an adaptogenic, antiphlogistic, antipyretic, antiseptic, aphrodisiac, febrifuge, stomachic, tonic, vermifuge, against asthma, bronchitis, scrofula, syphilis and in “controlling night emissions” and also to promote wound healing [19–21].

The phytochemical screening of *Evolvulus alsinoides* also showed the presence of glycosides, alkaloids, terpenoids, steroids, phenols and flavonoids in the ethanolic extract. Hence, the present study was aimed to evaluate the effect of ethanolic extract of *Evolvulus alsinoides* on insulin level, antioxidant status and lipid peroxidation in the pancreas of streptozotocin induced diabetic rats.

## Materials and methods

### Plant material

The whole plant of *Evolvulus alsinoides* (L.) L. used for the investigation was obtained from Coimbatore District, Tamilnadu, India. The plant was authenticated by Dr. P.Satyanarayana, Botanical Survey of India, TNAU Campus, Coimbatore. The voucher number is BSI/SRC/5/23/2011-12/Tech.-514. Fresh plant material was washed under running tap water, air dried and powdered.

### Sample extraction

100 g of dried plant powder was extracted in 500 ml of ethanol in an orbital shaker for 72 hrs. Repeated extraction was done with the same solvent till clear colorless solvent is obtained. Obtained extract was evaporated and stored at 0-4°C in an air tight container.

### Animals

Wistar albino rats weighing about 150–180 g were procured from Karpagam University Animal House, Coimbatore, India. The animals were under standard conditions and fed with rodent diet and water. The study was approved by Institutional Animal Ethical Committee constituted for the purpose of CPCSEA.

### Induction of experimental diabetes

Rats were rendered diabetic by a single intraperitoneal injection of freshly prepared streptozotocin at a dose of 45 mg/kg body weight in 0.1 M citrate buffer (pH 4.5) in a volume of 1 ml/kg body weight [22]. Diabetes was identified in rats by moderate polydypsia and marked polyuria. After 48 h of streptozotocin induction, blood glucose levels were estimated and rats with a blood glucose ranging between 200–400 mg/dl were considered diabetic and used for the experiments.

### Experimental protocol

The animals were divided into five groups of six animals each. Group I served as control; group II consisted of streptozotocin-induced diabetic rats; group III consisted of streptozotocin-induced diabetic rats treated with standard drug glibenclamide (1.25 mg/kg body weight/day/rat); groups IV consisted of streptozotocin-induced diabetic rats treated with whole plant ethanolic extract of *Evolvulus alsinoides* at a dose of 150 mg/kg body weight/day/rat and group V were normal rats treated with ethanolic extract of *Evolvulus alsinoides* (150 mg/kg body weight/day /rat).

### Biochemical studies

After 45 days of treatment the animals were sacrificed under chloroform anesthesia. The blood was collected; plasma sample was used for insulin and C-peptide estimation by using kit method. Pancreas was quickly excised off, a portion of pancreas washed with saline and the homogenate was prepared using 0.1 M phosphate buffer, pH 7.4. The homogenate was centrifuged and the supernatant was used for the determination of lipid peroxidation [23], enzymatic antioxidant like superoxide dismutase [24], catalase [25], glutathione peroxidase [26], glutathione reductase [27], glucose 6 phosphate dehydrogenase [28] and non-enzymatic antioxidants like total reduced glutathione [29], vitamin C [30] and vitamin E [31].

### Histological observation

One portion of pancreas of all the experimental groups was fixed in 10% formalin for histological observation by using the method of Dunn, 1974 [32].

### Statistical analysis

The values were expressed as Mean±SD. The statistical analysis was carried out by one way analysis of variance using SPSS (version 10) statistical analysis program. Statistical significance was considered at p < 0.05.

## Results

Diabetes mellitus (DM) is a serious health problem results from abnormality of carbohydrate metabolism and characterized by absolute (type І) or relative (type ІІ) deficiencies in insulin secretion or receptor insensitivity to endogenous insulin, resulting in hyperglycemia. DM affects various organs in the body and it is responsible for many complications like diabetic neuropathy, nephropathy and retinopathy. Hence the present study was undertaken to find out the therapeutic effect of *Evolvulus alsinoides* on antioxidants level in pancreas of streptozotocin induced diabetic rats.

Table 1 shows the effect of *Evolvulus alsinoides* on insulin and c-peptide levels of streptozotocin induced diabetic rats. Results showed a significant (p < 0.05) reduction in plasma insulin and C-peptide levels in streptozotocin induced rats. After treatment with whole plant ethanolic extract of *Evolvulus alsinoides* and glibenclamide for 45 days the values were reached near normal level as to that of control rats. The enzymatic antioxidant SOD, CAT, glutathione reductase, glucose 6 phosphate dehydrogenase and GPx levels were analyzed in control and streptozotocin induced rats and the results were represented in Table 2.

**Table 1:** **Effect of ethanolic extract of** ***Evolvulus alsinoides*** **on insulin and C-peptide in plasma of control and experimental groups**

Groups	Plasma Insulin (μU/ml)	C-Peptide (ng/ml)
**Control**	17.85±0.55^d^	6.28±0.27^d^
**Diabetic control**	6.8±0.54^a^	3.38±0.28^a^
**Diabetic + Glibenclamide**	15.78±0.46^c^	5.63±0.44^c^
**Diabetic +** ***Evolvulus alsinoides***	13.67±0.41^b^	4.75±0.18^b^
***Evolvulus alsinoides*** **alone**	16.3±0.42^c^	6.27±0.32^d^

Values are expressed as mean ± SD for six animals. Values not sharing common Superscript letters (a-f) differ significantly at p < 0.05 (DMRT).

**Table 2:** **Effect of ethanolic extract of** ***Evolvulus alsinoides*** **on enzymatic antioxidants in pancreas of diabetic induced and treatment groups**

Groups	SOD	Catalase	Glutathione reductase	GPx	Glucose-6-phosphate dehydrogenase
**Control**	3.00±0.81^b^	0.68±0.06^b^	1.88±0.25^c^	1.65±0.32^c^	2.87±0.08^b^
**Diabetic control**	1.14±0.64^a^	0.26±0.14^a^	0.83±0.39^a^	1.00±0.08^a^	1.75±0.52^a^
**Diabetic + Glibenclamide**	2.99±0.43^b^	0.62±0.07^b^	1.39±0.29b^c^	1.47±0.14^bc^	2.71±0.12^b^
**Diabetic +** ***Evolvulus alsinoides***	2.89±1.20^b^	0.62±0.05^b^	1.33±0.44^b^	1.27±0.12^ab^	2.85±0.21^b^
***Evolvulus alsinoides*** **alone**	3.34±0.45^b^	0.68±0.06^b^	1.61±0.56^c^	1.63±0.29^c^	2.98±0.09^b^

Values are expressed as mean ± SD for six animals in each group. Values not sharing common superscript letters (a-d) differ significantly at p < 0.05 (DMRT). Units: Superoxide dismutase - Enzyme required for 50% inhibition of NBT reduction/min/mg protein; Catalase - μmoles of H_2_O_2_utilized/min/mg/protein; Glutathione peroxidase - μmoles of GSH utilized/min/mg/protein; Glutathione reductase - n moles of NADPH oxidized/min/mg protein; Glucose 6 phosphate dehydrogenase - min/mg protein.

Table 3 shows the low levels of non-enzymatic antioxidant vitamin C, vitamin E and reduced glutathione in control and experimental group rats. In that the diabetic control group rats showed decreased levels of non-enzymatic antioxidants when compared to that of control rats. The levels of these antioxidants were significantly increased in diabetic rats by treating with plant extract.

**Table 3:** **Effect of ethanolic extract of** ***Evolvulus alsinoides*** **on non-enzymatic antioxidants in pancreas**

Groups	Total reduced glutathione	Vitamin C	Vitamin E
Control	25.38±1.10^c^	2.50±0.23^c^	2.78±0.42^c^
Diabetic control	16.61±2.08^a^	1.29±0.03^a^	1.21±0.49^a^
Diabetic + Glibenclamide	22.59±5.62^bc^	2.01±0.04^b^	1.93±0.36^b^
Diabetic + *Evolvulus alsinoides*	20.81±0.75^b^	2.12±0.12^b^	2.34±0.37^bc^
*Evolvulus alsinoides* alone	25.75±0.63^c^	2.55±0.02^c^	2.78±0.23^c^

Values are expressed as mean ± SD for six animals in each group. Values not sharing common superscript letters (a-d) differ significantly at p < 0.05 (DMRT).The units were expressed as μg/mg protein.

**Figure 1:**
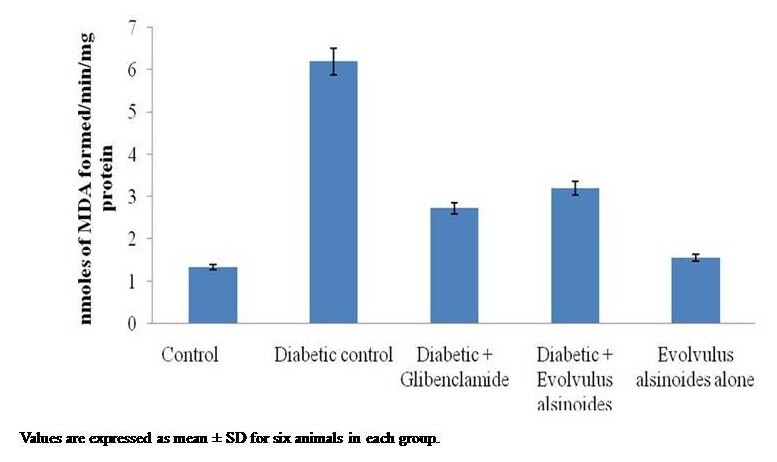
**Effect of** ***Evolvulus alsinoides*** **on lipid peroxidation in pancreas of control and diabetic rats.**

**Figure 2:**
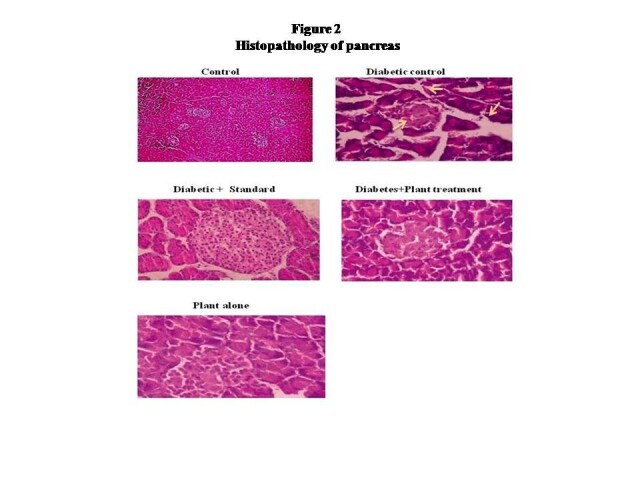
**Histopathology of pancreas.**

Figure 1 shows a statistically significant increase in lipid peroxide levels in streptozotocin induced diabetic rats with respect to normal controls and there was a significant decrease in lipid peroxide levels in diabetic rats treated with plant extract and standard drug glibenclamide. The histopathology of rat pancreas was shown in Figure 2. In that, the diabetic control rats showed complete destruction of pancreatic β-cell due to the induction of streptozotocin when compared to control. After administration of *Evolvulus alsinoides* and glibenclamide showed an increase in β-cell count and the remodeling of the structure of pancreas. The plant alone treated rats showed the normal β-cell indicates that the *Evolvulus alsinoides* did not cause any damage to pancreas.

## Discussion

Diabetes mellitus is a clinical syndrome characterized by inappropriate hyperglycemia caused by a relative or absolute deficiency of insulin or by a resistance to the action of insulin at the cellular level [33]. Streptozotocin induced hyperglycaemia has been described as a useful experimental model to study the effect of antidiabetic agents against diabetes mellitus. Streptozotocin enters the β cell via GLUT2 transporter and causes alkylation of DNA thereby induces the activation of poly ADP-ribosylation (a process that is more important for the diabetogenicity of streptozotocin than DNA damage itself). Poly ADP-ribosylation leads to depletion of cellular NAD^+^ and ATP. Enhanced ATP dephosphorylation after streptozotocin treatment supplies a substrate for xanthine oxidase resulting in the formation free radicals. Furthermore, streptozotocin liberates toxic amounts of nitric oxide that inhibits aconitase activity and participates in DNA damage. As a result of the streptozotocin action, β cells undergo the destruction by necrosis. Streptozotocin action in β cells is accompanied by characteristic alterations in blood insulin and glucose concentrations [34].

Antioxidants are substances or nutrients in our food substances which can avert or slow down the oxidative damage by quenching the free radicals in our body. When our body cells use oxygen, they naturally produce by-products (free radicals) which can cause damage to membranes and tissues. Health problems such as heart disease, muscular degeneration, diabetes mellitus, cancer etc. are contributed by oxidative damage [35].

C-peptide has insulin-mimetic effects on its own by activating insulin receptors, amino acid uptake and increases glycogen synthesis. The C-peptide promotes insulin action at low concentrations and inhibits it at high levels, suggesting a modulatory effect by C-peptide on insulin signaling [36]. The increased level of insulin and C-peptide was observed after treatment with ethanolic extract of *Evolvulus alsinoides* which may be due to the activation of trace β-cells in the pancreas. Our results are also in line with the previous report of Senthilkumar and Subramanian, 2008 [37]. Our results were also supported by Patel *et al*., 2009 [38] who reported that the diabetic induction by streptozotocin decreases the insulin level in serum, after treatment with dihar (poly herbal formation) the insulin levels were increased.

Oxidative stress is found to be increased in patients with diabetes mellitus. Evidence suggests that oxidative cellular injury caused by free radicals contributes to the development of diabetes. Antioxidant enzymes are used to scavenge the free radicals and protects the organs and membranes from oxidative damage. Moreover, diabetes also induces changes in the tissue content and activity of the antioxidant enzymes [39, 40]. SOD protects tissues against oxygen free radicals by catalysing the removal of superox-ide radical, converting it into H_2_O_2_ and molecular oxygen, which both damage the cell membrane and other biological structures [41]. Catalase is a haem-protein, which is responsible for the detoxification of significant amounts of H_2_O_2_[42]. Reduced activities of SOD and catalase in the liver and pancreas during diabetes were reported, resulting in a number of deleterious effects due to the accumulation of superoxide radicals and hydrogen peroxide [43]. The plant extract treated rats showed reduced lipid peroxidation which was associated with an increased activity of antioxidant enzymes like SOD and CAT. The same results was reported by Chis *et al.,* 2009 [1] for antioxidant effects of a grape seed extract in a rat model of diabetes mellitus.

In our study, decline in the activities of these enzymes in STZ induced animals and attained near to normal in plant treated rats indicates oxidative stress elicited by STZ had been nullified due to the effect of the extract. Similar to the finding in this study, a decrease has been observed in the activities of GST, CAT and GPx in some of the tissues of diabetic rats [35, 44].

Reduced glutathione is one of the most abundant non-enzymatic antioxidant bio-molecules present in tissues whose functions are removal of reactive oxygen species and provision of a substrate for GPx and glutathione S-transferase (GST) [45]. Ascorbic acid is most powerful antioxidant under physiological conditions. It can directly scavenge superoxide, hydroxyl radicals and single oxygen [46]. The most important antioxidant in the cell membrane is alpha tocopherol. It interrupts the chain reaction of lipid peroxidation by reacting with lipid peroxyl damage [47]. After treatment with plant extract increased level of alpha tocopherol found in the STZ diabetic rats which may be due to the release of membrane bound alpha tocopherol from damaged cell membrane.

Lipid peroxidation is a characteristic of diabetes mellitus. The increase of free radicals in diabetic condition is suggested to be due to the increased lipid peroxidation and the damage of antioxidant defense system [15]. Reactive oxygen metabolites have been implicated in the damage brought by ionizing radiation, as well as in the effects of several cytostatic compounds. The decreased activity of antioxidant molecules along with elevated lipid peroxide levels in diabetic rats could probably be associated with oxidative stress and decreased antioxidant defense potential [48]. Our results showed that in diabetic animals the levels of lipid peroxidation were high in pancreas and were restored to normal values after the treatment with plant extract. These antioxidant effects may be due to the presence of presence of secondary metabolites like phenols, flavonoids, alkaloids, steroids, terpenoids and glycosides in the ethanolic extract of *Evolvulus alsinoides* (L.) L [49, 50].

Histological studies were carried out over the pancreas for the five groups i.e. control, diabetic control, diabetic treated with standard, diabetic treated plant extract and plant extract alone. In that treated two groups exposed the capability restoring the organ to normal histology when compared to the morphological disruptions in the diabetic control rat. The results obtained as shown in Figure 2 revealed the inhibitory and protective effect of plant extract over the organ damages at hyperglycemic conditions.

## Conclusions

The present study showed that *Evolvulus alsinoides* extract reduces the lipid peroxidation level and increases the antioxidant level in experimental rats. It also prevents the pancreas by suppressing the oxidative stress in associated with diabetes and also help to increase the insulin level by remodeling the structure of pancreas. Although the exact chemical compounds responsible for the hypoglycemic effects of *Evolvulus alsinoides* still re-main exploratory; experimental evidence obtained from this study indicates that this plant has an antioxidant activity and improves the insulin secretion from pancreas.

## Author’s contribution

DG participated in the study design, statistical analysis, data interpretation, writing and alignment of this manuscript. GR participated in the practical part of this work. KM participated in both practical as well as statistical analysis part of this work. KD participated in the interpretation and correction of this manuscript. CU contributed in the study design, interpretation and also in the correction of the manuscript. All authors read and approved the final manuscript.

## Authors’ original submitted files for images

Below are the links to the authors’ original submitted files for images. Authors’ original file for figure 1 Authors’ original file for figure 2
