# Supplementary figures and images for: Efficacy of Evolvulus alsinoides (L.) L. on insulin and antioxidants activity in pancreas of streptozotocin induced diabetic rats
Source: J Diabetes Metab Disord. 2013 Jul 8;12:39. doi: 10.1186/2251-6581-12-39 (PMC7983677; doi:10.1186/2251-6581-12-39)

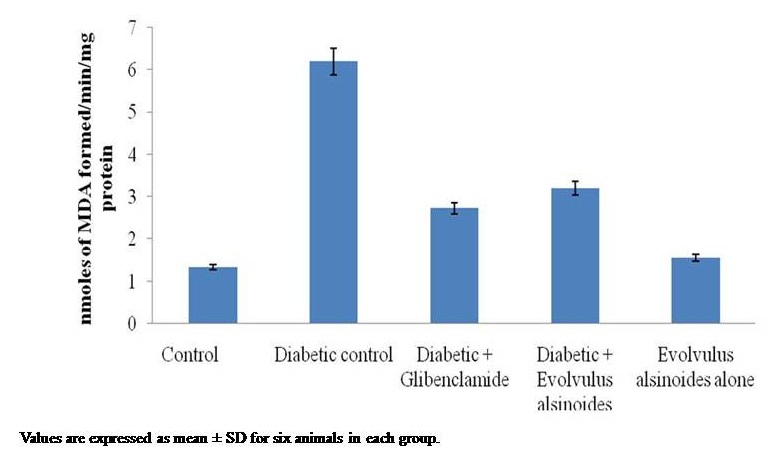

Supplement: Supplementary file 1 — Authors’ original file for figure 1 [file 40200_2013_63_MOESM1_ESM.jpeg]

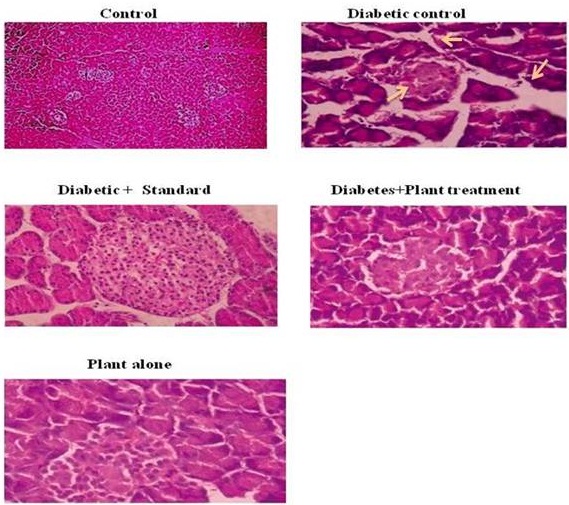

Supplement: Supplementary file 2 — Authors’ original file for figure 2 [file 40200_2013_63_MOESM2_ESM.jpeg]
